# ATG5 negatively regulates grass carp reovirus-induced immune-inflammatory response by degrading RIG-I and MDA5

**DOI:** 10.1128/jvi.00344-25

**Published:** 2025-06-02

**Authors:** Pengfei Chu, Yingying Wang, Jian Shen, Yaping Wang, Guobin Chang, Libo He

**Affiliations:** 1 College of Animal Science and Technology, Yangzhou University 614678 , Yangzhou, China; 2 State Key Laboratory of Breeding Biotechnology and Sustainable Aquaculture, Institute of Hydrobiology, Chinese Academy of Sciences 53021 , Wuhan, China; 3 Innovative Academy of Seed Design, Chinese Academy of Sciences, Beijing, China; 4 University of Chinese Academy of Sciences, Beijing, China; University of Michigan Medical School, Ann Arbor, Michigan, USA

**Keywords:** ATG5, grass carp reovirus, immune-inflammatory response, degradation, RIG-I, MDA5, IRF7, phosphorylation

## Abstract

Grass carp reovirus (GCRV) is the most virulent aquareovirus, inducing hemorrhagic disease that results in significant economic losses for the grass carp aquaculture industry in China. It has been reported that the cytokine storm, resulting from the viral-induced acute immune-inflammatory response, is the primary cause of death associated with viral infections. Therefore, exploring the negative regulatory factors of the immune response may provide important targets for virus prevention and control. In this study, we demonstrated that GCRV infection induces an acute immune-inflammatory response in yearling grass carp, while a milder immune response is observed in 3-year-old grass carp by assessing key gene expression at both the mRNA and protein levels. Furthermore, we found that the upregulation of autophagy core gene, autophagy-related gene 5 (ATG5), inhibits GCRV replication and reduces the excessive immune-inflammatory response induced by the virus. Notably, co-immunoprecipitation and bimolecular fluorescence complementation reveal that ATG5 interacts with RIG-I and MDA5, inhibiting the downstream immune response. Mechanistically, ATG5 promotes autophagy and K48-linked polyubiquitination to degrade RIG-I and MDA5. Importantly, ATG5 reduces IRF7 phosphorylation through the degradation of RIG-I and MDA5, which, in turn, leads to a decreased immune response during GCRV infection. Collectively, our findings suggest that ATG5 serves as an important negative regulatory factor of the viral-induced acute immune-inflammatory response, providing a promising target for the prevention and control of GCRV.

**IMPORTANCE:**

Grass carp reovirus (GCRV) is a highly virulent pathogen responsible for hemorrhagic disease in grass carp, leading to devastating losses in aquaculture. The study reveals that GCRV triggers an acute immune-inflammatory response in young grass carp, which is linked to high mortality, while a milder immune response occurs in older, resistant fish. Importantly, the autophagy-related gene ATG5 was identified as a critical regulator that suppresses excessive immune-inflammatory responses by promoting the degradation of RIG-I and MDA5, two key viral sensors. ATG5 not only reduces viral replication but also limits IRF7 phosphorylation, a key step in interferon signaling. This research uncovers a novel mechanism by which autophagy moderates viral-induced immune response, suggesting that ATG5 could be a valuable target for therapeutic interventions aimed at controlling GCRV infection and preventing cytokine storms in aquaculture. These findings could have broader implications for managing viral infections in fish farming.

## INTRODUCTION

Type I interferons (IFN-I) are a critical component of the innate immune response to viral infections, inducing a range of antiviral gene expressions (1–3). This response, one of the first lines of host defense against invading pathogens, is precisely controlled by various regulatory molecules (4, 5). IFN-positive regulators enhance the antiviral immune response and clear viral infections, while IFN-negative regulators dampen inflammatory responses to prevent immune-mediated tissue damage and spontaneous autoimmunity (6, 7). Therefore, dynamic and effective early innate immune responses are crucial for determining the clinical outcomes of viral infections. If these responses are dysregulated, immune-inflammatory dysregulation may facilitate viral infection and replication, leading to immune-mediated tissue damage (8).

In the context of aquaculture, understanding these immune responses is particularly crucial for species like grass carp (*Ctenopharyngodon idellus*), which contributes over 21% to China’s total freshwater aquaculture production. In 2023, grass carp production reached 5.94 million tons, establishing it as the most widely consumed freshwater fish in China (9). Despite its importance, grass carp is highly vulnerable to various pathogens, particularly grass carp reovirus (GCRV), which causes grass carp hemorrhagic disease and poses a major challenge for grass carp aquaculture (10, 11). As a result, GCRV has become a focal point for fish breeding scientists and immunologists who are working to develop disease-resistant strains and to better understand the mechanisms of GCRV infection (12–16).

Although innate immune responses, such as IFN, are synthesized and secreted following viral infection, mortality still frequently occurs. For instance, SARS-CoV-2 infection triggers an acute immune response in COVID-19 patients, yet some patients still succumbed to the disease, especially in the early stages of the pandemic (17). Similarly, GCRV infection in grass carp was observed to induce an acute immune-inflammatory response and complement activation, yet it resulted in more than 80% mortality in the affected fish (18). These findings suggest that virus-induced acute immune responses may be ineffective or dysregulated. It has been reported that a cytokine storm, resulting from an ineffective or dysregulated immune response, is a significant cause of mortality (19). Therefore, identifying negative regulatory factors of the immune response could provide crucial targets for viral prevention and control.

In this study, we identified the grass carp autophagy-related gene 5 (ATG5) as a negative regulator of the acute immune-inflammatory response induced by GCRV. We demonstrated that GCRV infection triggers an acute immune-inflammatory response in yearling grass carp, which plays a crucial role in determining the infection outcome. ATG5 inhibits GCRV replication and reduces the excessive immune-inflammatory response triggered by the virus. It interacts with RIG-I and MDA5, promoting autophagy and K48-linked polyubiquitination, leading to the degradation of RIG-I and MDA5. This degradation results in decreased IRF7 phosphorylation, thereby reducing the immune response during GCRV infection. Our findings suggest that targeting ATG5 may be a promising strategy for the prevention and control of GCRV.

## RESULTS

### GCRV infection induces acute immune-inflammatory response in yearling grass carp

**Fig 1:**
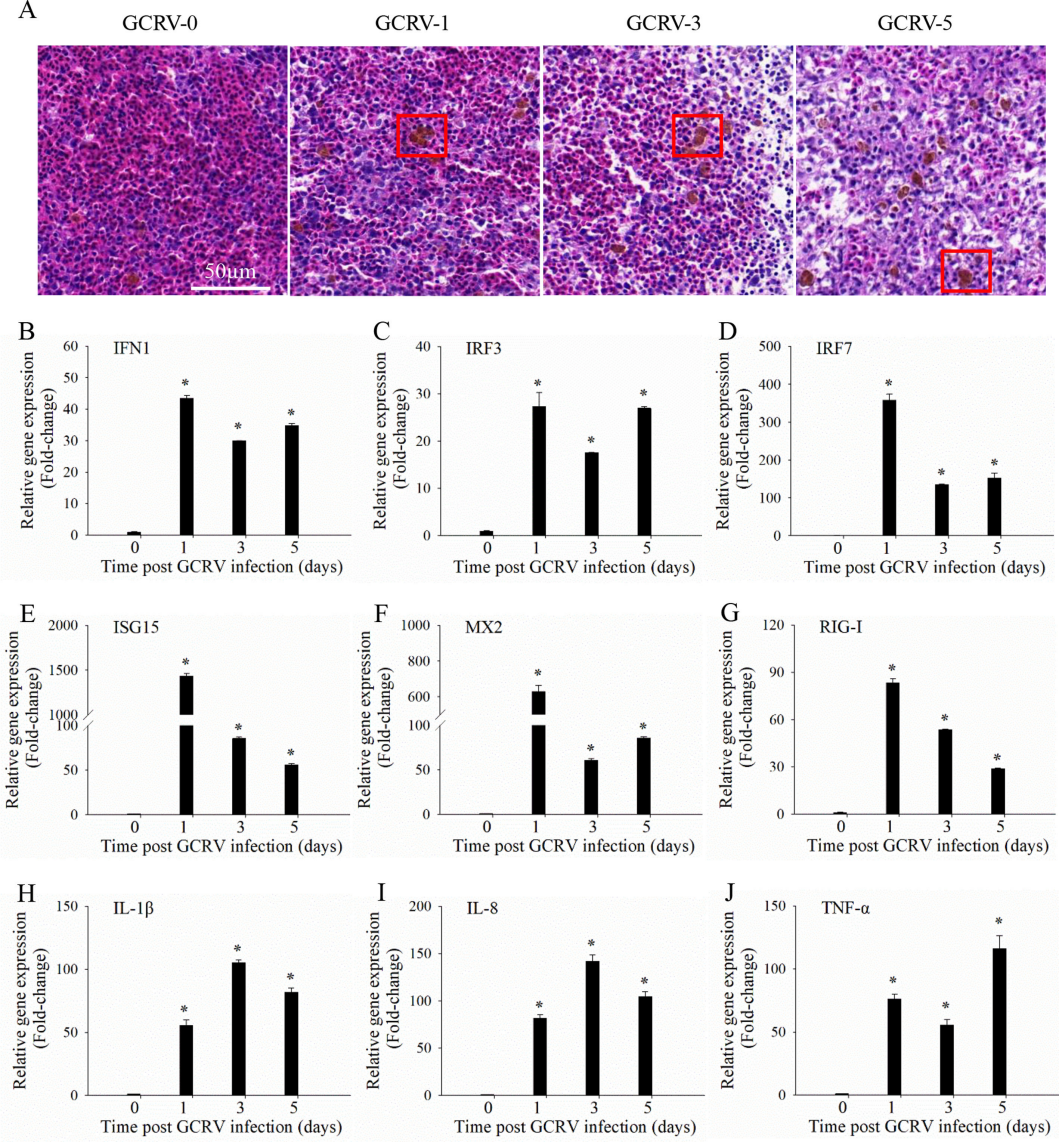
GCRV infection induces acute immune-inflammatory response in yearling grass carp. (**A**) Histological section analysis of spleen samples from yearling grass carp after GCRV infection. Yearling grass carp were infected with GCRV, and spleen samples were collected on 0, 1, 3, and 5 days (GCRV-0, 1, 3, and 5) post-infection for histological section analysis. The red box indicated eosinophils. Scale bars = 50 µm. (B–J) RT-qPCR analysis of the mRNA expression levels of immune-inflammatory genes in yearling grass carp after GCRV infection. Yearling grass carp were infected with GCRV, and the mRNA expression of IFN-1, IRF3, IRF7, ISG15, MX2, RIG-I, IL-1β, IL-8, and TNF-α in the spleen samples was analyzed. Data are represented as mean (*n* = 3) ±SD. * indicates *P* < 0.05.

Precise and effective immune responses are crucial for defending against pathogen infections. In contrast, dysregulated immune responses can lead to autoinflammation and severe tissue damage (20). To investigate the host immune response during GCRV infection *in vivo*, yearling grass carp were infected with GCRV, and the spleen samples were collected at different time points for analysis. As shown in Fig. 1, histological sections revealed that GCRV infection caused significant tissue damage, including vacuolization, nuclear karyorrhexis, and severe necrotic lesions in the spleen samples (Fig. 1A). Furthermore, eosinophils, which are closely associated with immune response and inflammation (21), were rapidly induced and accumulated following GCRV infection (Fig. 1A). Additionally, we examined the expression levels of immune-related genes using RT-qPCR. The results showed that IFN1 was rapidly induced and persistently upregulated following GCRV infection (Fig. 1B). Similar expression patterns were observed for other IFN-related genes, such as IRF3, IRF7, RIG-I, ISG15, and MX2, as well as inflammation factors like IL-1β, IL-8, and TNF-α (Fig. 1C through J). These findings suggest that GCRV infection triggers an acute immune-inflammatory response, along with severe tissue damage in yearling grass carp.

### Acute immune-inflammatory response is associated with the outcomes of GCRV infection

**Fig 2:**
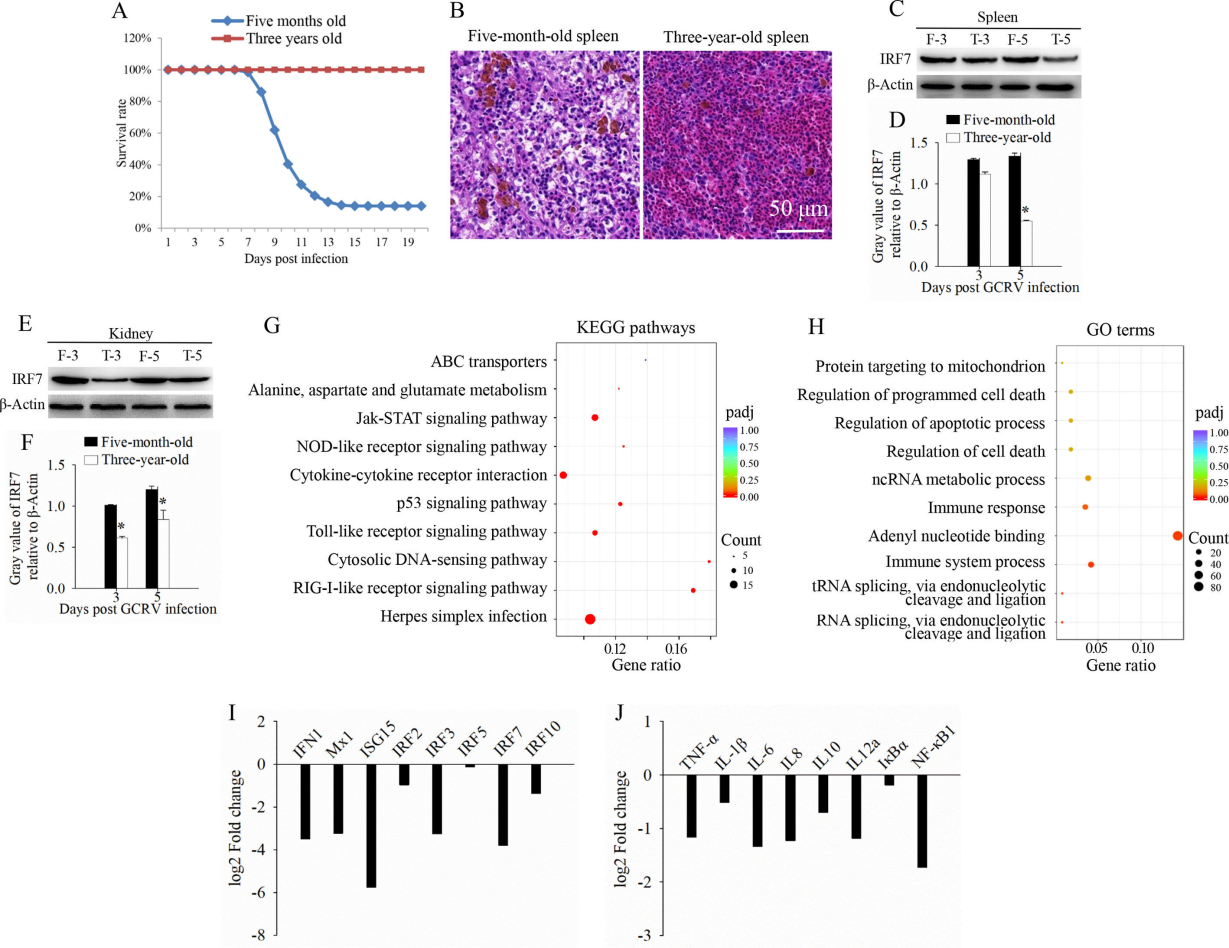
Acute immune-inflammatory is associated with the outcomes of GCRV infection. (**A**) Percent of survival in 5-month-old and 3-year-old grass carp groups. A viral challenge experiment was conducted for 300 five-month and 300 three-year grass carp, and the percentage of survival was calculated. (**B**) Histological section analysis of spleen samples from 5-month-old and 3-year-old grass carp at 5 days post-GCRV infection. Scale bars = 50 µm. (C–F) Protein expression level of IRF7 in two fish groups after GCRV infection. Five-month-old and 3-year-old grass carp were infected with GCRV, and spleen (C and D) and kidney (E and F) tissues were collected on 3 and 5 days post-infection to detect the protein expression levels (**C, E**) and calculate the gray values (**D, F**) of IRF7. (**G**) The top 10 enriched KEGG terms for the downregulated DEGs between two fish groups after GCRV infection. (**H**) The top 10 enriched GO terms for the downregulated DEGs between two fish groups after GCRV infection. (**I**) Expression patterns of interferon-related genes between two fish groups after GCRV infection. (**J**) Expression patterns of inflammatory cytokines between two fish groups after GCRV infection. Data are represented as mean (*n* = 3) ±SD. * indicates *P* < 0.05.

Although the results above revealed that GCRV infection induces a strong immune-inflammatory response *in vivo*, mortality was consistently observed in GCRV-infected fish, particularly in yearling grass carp. Our previous report showed that grass carp older than 3 years are completely resistant to GCRV infection (15), providing a natural model to investigate the pathogenesis of GCRV infection. Therefore, a viral challenge experiment was conducted on 5-month-old and 3-year-old grass carp. As shown in Fig. 2, all 3-year-old grass carp survived after GCRV infection, whereas the vast majority of 5-month-old grass carp died 15 days post-GCRV infection, with a survival rate of only 14% (Fig. 2A). Histological analysis revealed severe tissue damage and a large number of eosinophils in the 5-month-old grass carp, while only a few eosinophils were detected in the 3-year-old grass carp (Fig. 2B). Western blotting revealed the protein expression level of IRF7 in 3-year-old grass carp was significantly lower than that in 5-month-old grass carp (Fig. 2C through F). To gain a more comprehensive understanding of the gene expression patterns in the IFN-inflammatory axis, a transcriptome analysis was performed. The transcriptional profiles (GenBank accession number: PRJNA600033) from the 3-year-old fish group were compared to those of the 5-month-old fish group to identify differentially expressed genes (DEGs). KEGG enrichment analysis of DEGs showed that the downregulated genes were significantly enriched in immune response pathways, such as the NOD-like receptor signaling pathway, Toll-like receptor signaling pathway, and RIG-I-like receptor (RLR) signaling pathway (Fig. 2G). Consistent with these findings, GO enrichment analysis demonstrated that terms related to immune response and regulated cell death were also enriched in the downregulated DEGs following GCRV infection (Fig. 2H). Moreover, genes involved in the IFN signaling pathway, such as IFN1, Mx1, ISG15, IRF2, IRF3, IRF5, IRF7, and IRF10, along with inflammatory cytokines including TNF-α, IL-1β, IL-6, IL-8, IL-10, IL-12a, IκBα, and NF-κB1, were lower in the 3-year-old fish group compared to the 5-month-old fish group (Fig. 2I and J). Given that all 3-year-old grass carp survived while the vast majority of 5-month-old grass carp died following GCRV infection, it is evident that the acute immune-inflammatory response in 5-month-old grass carp is either ineffective or dysregulated. This acute immune-inflammatory response is strongly associated with the outcomes of GCRV infection.

### ATG5 reduces GCRV-induced immune response

**Fig 3:**
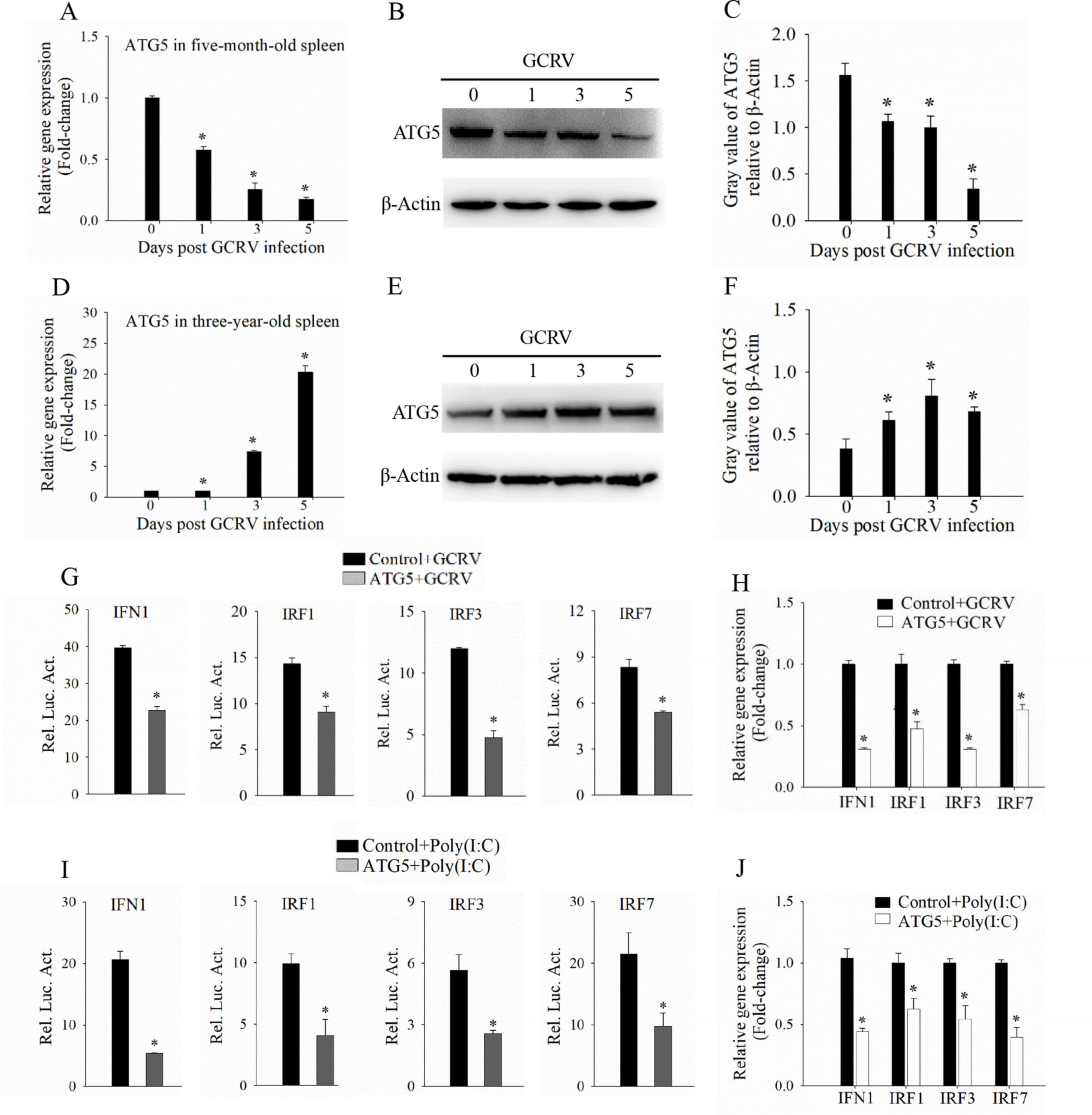
ATG5 reduces GCRV-induced immune response. (A–C) RT-qPCR and western blotting analysis of the expression level of ATG5 in 5-month-old grass carp following GCRV infection. The spleen samples of 5-month-old grass carp were sampled on 0, 1, 3, and 5 dpi to detect the mRNA and protein expression of ATG5. (D–F) RT-qPCR and western blotting analysis of the expression level of ATG5 in 3-year-old grass carp following GCRV infection. The spleen samples of 3-year-old grass carp were sampled on 0, 1, 3, and 5 dpi to detect the mRNA and protein expression of ATG5. (**G, H**) Analysis of the promoter activities and mRNA expression levels of interferon-related genes in ATG5- or empty vector-transfected cells after GCRV infection. (**I, J**) Analysis of the promoter activities and mRNA expression levels of interferon-related genes in ATG5- or empty vector-transfected cells after Poly(I:C) stimulation. Data are represented as mean (*n* = 3) ±SD. * indicates *P* < 0.05.

Since the acute disordered immune-inflammatory response is closely associated with the outcomes of GCRV infection, exploring the negative regulatory factors of the immune response may provide important targets for the prevention and control of GCRV. Our previous study demonstrated that autophagy inhibits GCRV replication and attenuates acute inflammatory responses to protect cells (13). Therefore, we investigated the expression level of an autophagy core gene, ATG5, in the two grass carp groups after GCRV infection. As shown in Fig. 3, the mRNA and protein expression level of ATG5 was decreasing in 5-month-old grass carp following GCRV infection (Fig. 3A through C). In contrast, ATG5 expression showed an opposite pattern in 3-year-old grass carp (Fig. 3D through F). These results suggest that the different expression pattern of ATG5 between the two differentiated-age grass carp may be associated with the host immune response after GCRV infection. Subsequently, we examined the role of ATG5 in regulating GCRV- and Poly(I:C)-induced IFN expression. As depicted in Fig. 3, ATG5 suppressed the promoter activities of key IFN-related genes, including IFN1, IRF1, IRF3, and IRF7, following GCRV infection (Fig. 3G). The RT-qPCR results also showed similar trends (Fig. 3H). Additionally, we also found that ATG5 significantly attenuated both the promoter activities and mRNA expression levels of IFN-related genes in response to Poly(I:C) stimulation (Fig. 3I and J). Collectively, these findings suggest that ATG5 reduces GCRV-induced immune response.

### ATG5 antagonizes GCRV replication and reduces excessive immune-inflammatory response

**Fig 4:**
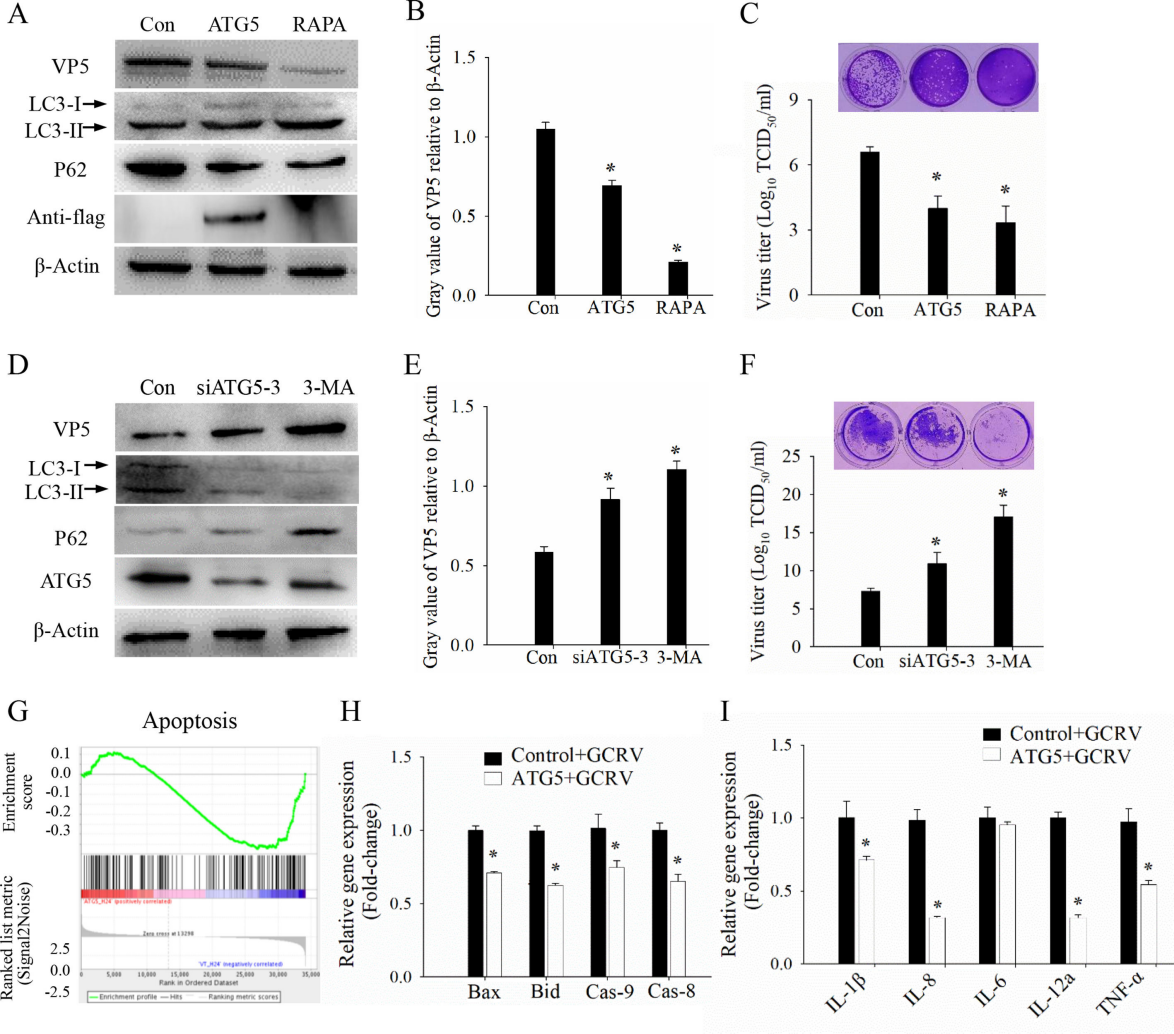
ATG5 antagonizes GCRV replication and reduces excessive immune-inflammatory response. (A–C) Investigation of the role of ATG5 in GCRV infection and autophagy regulation by overexpression. Cells were transfected with ATG5 or empty vector for 24 h, or treated with 100 nM Rapa for 6 h, followed by GCRV infection. After 24 h, cells were sampled for western blotting analysis (**A**), gray value calculation (**B**), and virus titer determination (**C**). (D–F) Investigation of the role of ATG5 in GCRV infection and autophagy regulation by siRNA. Cells were transfected with siRNA of ATG5 or control sequence for 24 h, or treated with 5 mM 3-MA for 6 h, followed by GCRV infection. After 24 h, cells were sampled for western blotting analysis (**D**), gray value calculation (**E**), and virus titer determination (**F**). (**G**) Gene set enrichment analysis (GSEA) of the downregulated DEGs in the apoptosis signaling pathway between ATG5- and empty vector-transfected cells after GCRV infection. (**H**) RT-qPCR analysis of the mRNA expression level of apoptosis-related genes in ATG5- or empty vector-transfected cells after GCRV infection. (**I**) RT-qPCR analysis the mRNA expression level of inflammatory cytokines in ATG5- or empty vector-transfected cells after GCRV infection. Data are represented as mean (*n* = 3) ±SD. * indicates *P* < 0.05.

The above results demonstrate that a dysregulated immune-inflammatory response occurs during GCRV infection, while ATG5 plays a role in mitigating the GCRV-induced immune response. To further investigate the role of ATG5 in GCRV replication, we examined its effect in cultured cells. Cells were transfected with ATG5 or an empty vector for 24 h, or treated with 100 nM rapamycin (Rapa) for 6 h, followed by GCRV infection. The results showed that ATG5-induced autophagy, along with Rapa treatment, significantly inhibited GCRV VP5 protein expression (Fig. 4A and B). Additionally, less cytopathic effect (CPE) induced by GCRV was observed, and viral titers were significantly reduced in the ATG5-transfected group compared to the control group (Fig. 4C). Moreover, cells were also transfected with ATG5-specific siRNA or a control sequence for 24 h, or treated with 5 mM 3-Methyladenine (3-MA) for 6 h, followed by GCRV infection. Conversely, reducing ATG5 mRNA levels using siRNA or treatment with 3-MA enhanced the expression of GCRV VP5 (Fig. 4D and E). Additionally, ATG5 knockdown resulted in increased CPE and viral titers (Fig. 4F). Transcriptome analysis (accession numbers: PRJNA597582 and PRJNA597542) was conducted on cells transfected with either ATG5 or empty vector, followed by GCRV infection. Gene set enrichment analysis (GSEA) results showed that the apoptosis signaling pathway was significantly enriched among the downregulated DEGs (Fig. 4G). RT-qPCR confirmed that the expression levels of pro-apoptotic genes, such as Bax, Bid, Caspase-8, and Caspase-9, as well as inflammatory cytokines, including IL-1β, IL-8, IL-6, IL-12a, and TNF-α, were lower in the ATG5-transfected group compared to the control group (Fig. 4H and I). These data indicate that ATG5 antagonizes GCRV replication and mitigates the excessive immune-inflammatory response.

### ATG5 interacts with RIG-I and MDA5 and inhibits their induced immune response

**Fig 5:**
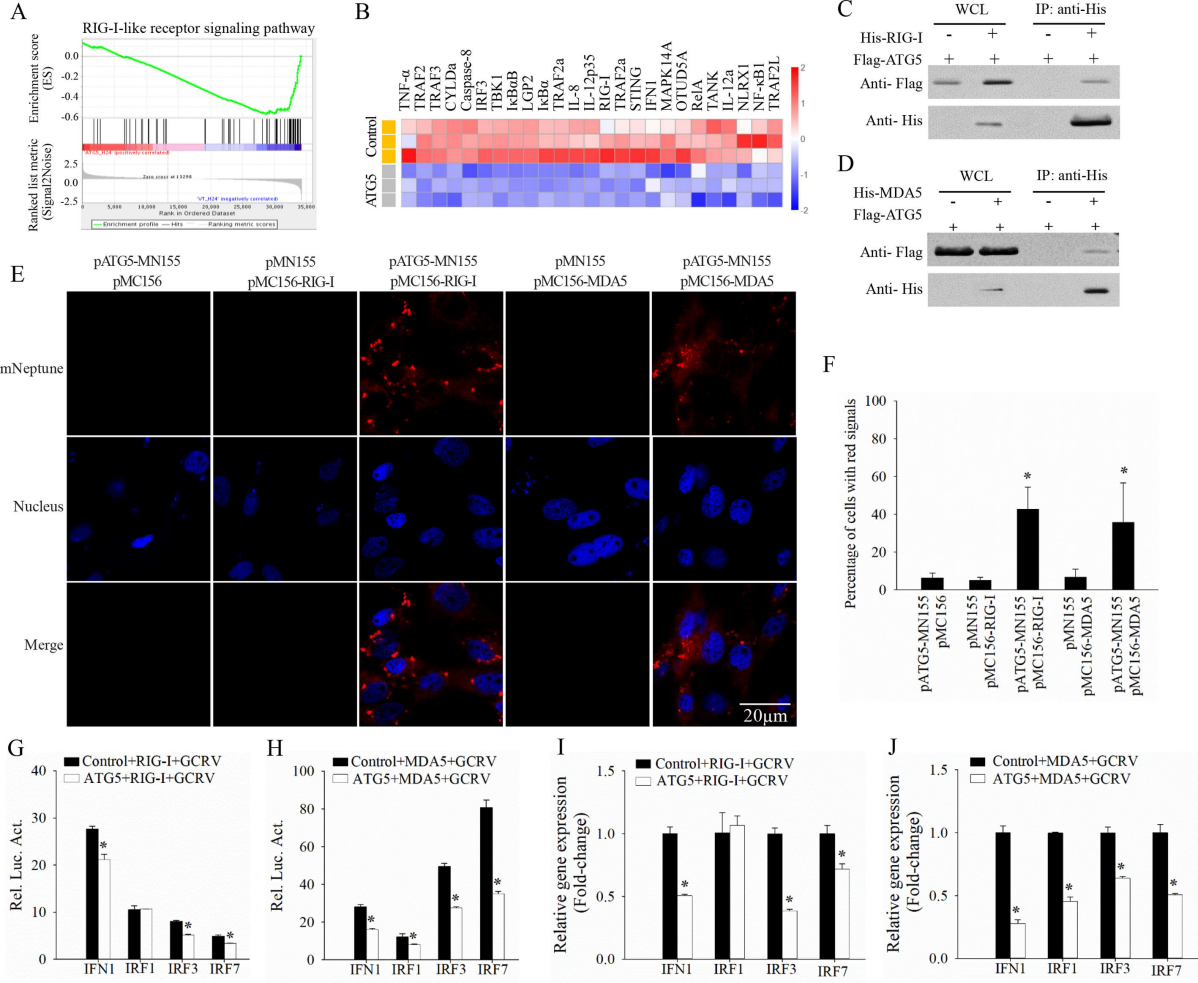
ATG5 interacts with RIG-I and MDA5 and inhibits their induced immune response. (**A**) Gene set enrichment analysis (GSEA) of the downregulated DEGs in RLR signaling pathway between ATG5- and empty vector-transfected cells after GCRV infection. (**B**) Heatmap of the top 25 downregulated DEGs in RLR signaling pathway between ATG5- and empty vector-transfected groups. (**C, D**) Co-immunoprecipitation analysis of the interaction between ATG5 and RIG-I (**C**) or MDA5 (**D**). (**E**) Far-red mNeptune-based BiFC analysis of the interaction between ATG5 and RIG-I or MDA5. Scale bars = 20 µm. (**F**) Quantitative analysis of the percentage of cells with red fluorescence signals of mNeptune in a field of view. (**G, H**) Analysis of the promoter activities of key interferon-related genes in ATG5- or empty vector-transfected cells after RIG-I/ MDA5 induction and GCRV infection. (**I, J**) Analysis of the mRNA expression of key interferon-related genes in ATG5- or empty vector-transfected cells after RIG-I/MDA5 induction and GCRV infection. Data are represented as mean (*n* = 3) ±SD. * indicates *P* < 0.05.

We further analyzed the transcriptome data from cells transfected with either ATG5 or empty vector, followed by GCRV infection. GSEA enrichment analysis based on these DEGs revealed that the RLR signaling pathway was significantly downregulated in the ATG5 overexpression group (Fig. 5A and B). Next, we explored the interactions between ATG5 and RLRs using co-immunoprecipitation (Co-IP) and far-red mNeptune-based bimolecular fluorescence complementation (BiFC). CIK cells were co-transfected with Flag-tagged ATG5 and His-tagged RIG-I or MDA5 and then subjected to Co-IP analysis. The results showed that ATG5 can bind to RIG-I and MDA5 (Fig. 5C and D). Furthermore, pMN155-ATG5 was co-transfected with pMC156-RIG-I or pMC156-MDA5, and fluorescence observation was performed. The results demonstrated that red fluorescence signals of mNeptune were observed in cells co-transfected with ATG5 and either RIG-I or MDA5 (Fig. 5E and F), but neither in the corresponding controls, further confirming that ATG5 interacts specifically with RIG-I and MDA5. Additionally, we investigated the influence of ATG5 on the immune response induced by RIG-I or MDA5. The results indicated that ATG5 inhibited the promoter activities of key genes during GCRV infection, including IFN1, IRF1, IRF3, and IRF7, which were induced by RIG-I or MDA5 (Fig. 5G and H). Consistently, RT-qPCR results showed that ATG5 significantly suppressed the transcription of these genes induced by RIG-I or MDA5 (Fig. 5I and J). Collectively, these findings demonstrate that ATG5 inhibits the immune response induced by RIG-I and MDA5.

### ATG5 promotes K48-linked polyubiquitination to degrade RIG-I and MDA5

**Fig 6:**
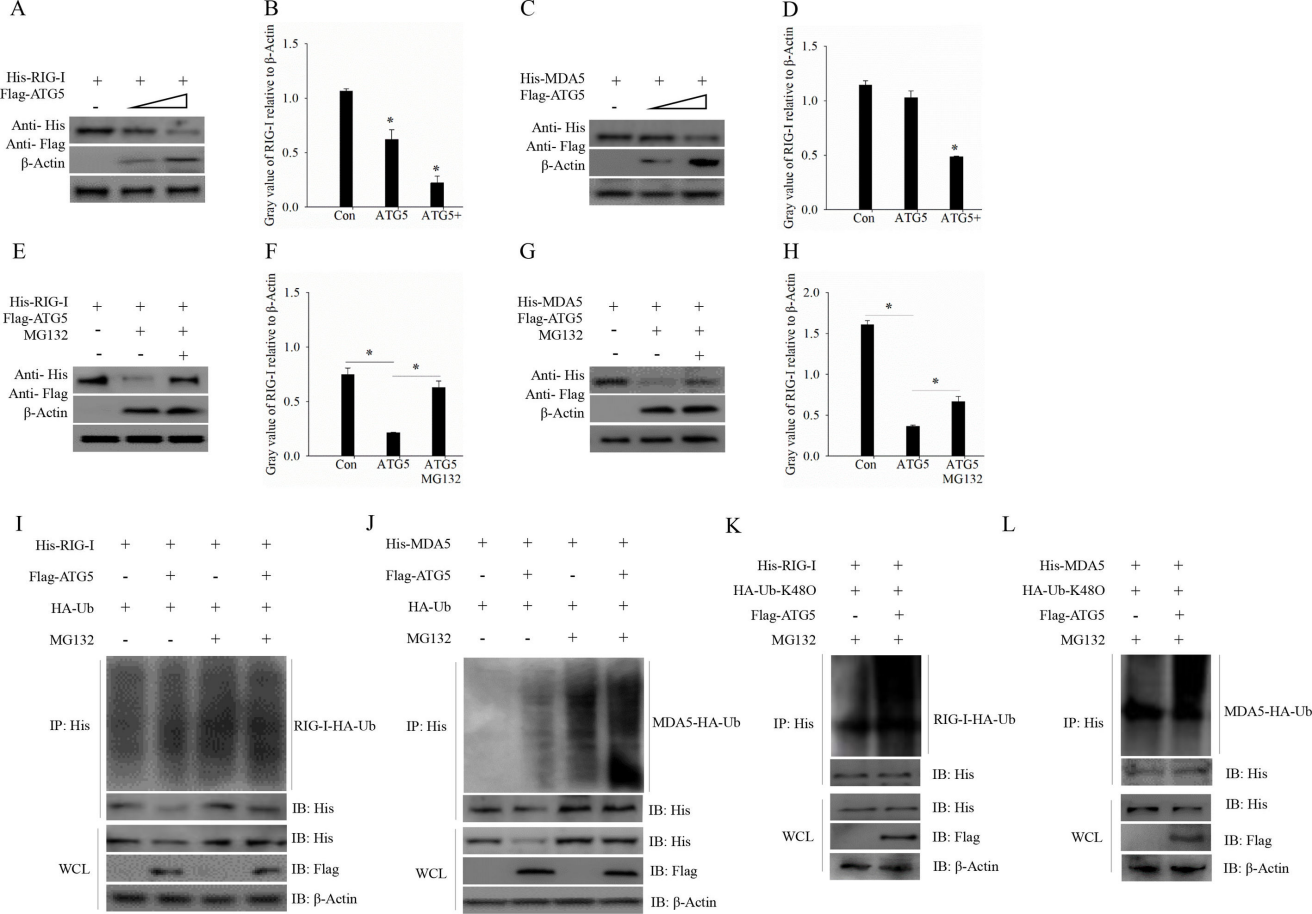
ATG5 promotes K48-linked polyubiquitination to degrade RIG-I and MDA5. (A–D) ATG5 degrades RIG-I (**A, B**) and MDA5 (**C, D**) in a dose-dependent manner. Different doses of ATG5 were co-transfected with RIG-I or MDA5 into cells for 24 h and then harvested for western blotting analysis (**A, C**) and gray value calculation (**B, D**). (E–H) MG132 blocked ATG5-mediated degradation of RIG-I (**E, F**) and MDA5 (**G, H**). ATG5 was co-transfected with RIG-I or MDA5 into cells in the absence or presence of MG132 and then harvested for western blotting analysis (**E, G**) and gray value calculation (**F, H**). (**I, J**) ATG5 promotes the ubiquitination of RIG-I (**I**) and MDA5 (**J**). Cells co-transfected with ATG5, RIG-I or MDA5, and ubiquitin (Ub) in the absence or presence of MG132 and then harvested for IP and western blotting analysis. (**K, L**) ATG5 mediates the K48-linked ubiquitination of RIG-I (**K**) and MDA5 (**L**). Cells co-transfected with ATG5, RIG-I or MDA5, and K48-linked ubiquitin in the absence or presence of MG132 and then harvested for IP western blotting analysis.

To further investigate the molecular mechanism by which ATG5 inhibits RIG-I and MDA5-induced immune responses, we first assessed the effect of ATG5 on these proteins at the protein level. As shown in Fig. 6, ATG5 overexpression resulted in the degradation of RIG-I and MDA5 in a dose-dependent manner (Fig. 6A through D). It has been reported that ubiquitination modification is a classical mechanism of protein degradation that can be disrupted by the proteasome inhibitor MG132 (22, 23). Therefore, we examined whether ATG5-mediated degradation of RIG-I and MDA5 depends on the proteasome pathway. As expected, ATG5 overexpression significantly reduced the levels of RIG-I and MDA5, whereas their protein levels were partially restored in the presence of MG132 (Fig. 6E through H), indicating that the degradation of RIG-I and MDA5 by ATG5 is a proteasome-dependent process. Moreover, we transfected RIG-I, MDA5, ubiquitin (Ub), and ATG5 into cells in the presence or absence of MG132 and subsequently performed WB analysis. The results revealed that ATG5 enhanced the ubiquitination of both RIG-I and MDA5 (Fig. 6I and J). Since K48-linked polyubiquitin chain modification typically leads to proteasome recognition and degradation of the target protein (22), K48-linked ubiquitination assays were conducted. As anticipated, ATG5 promoted the K48-linked polyubiquitination of RIG-I and MDA5 (Fig. 6K and L). Collectively, these results suggest that ATG5 promotes K48-linked polyubiquitination of RIG-I and MDA5, leading to their degradation via a proteasome-dependent pathway.

### ATG5 promotes autophagy to degrade RIG-I and MDA5

**Fig 7:**
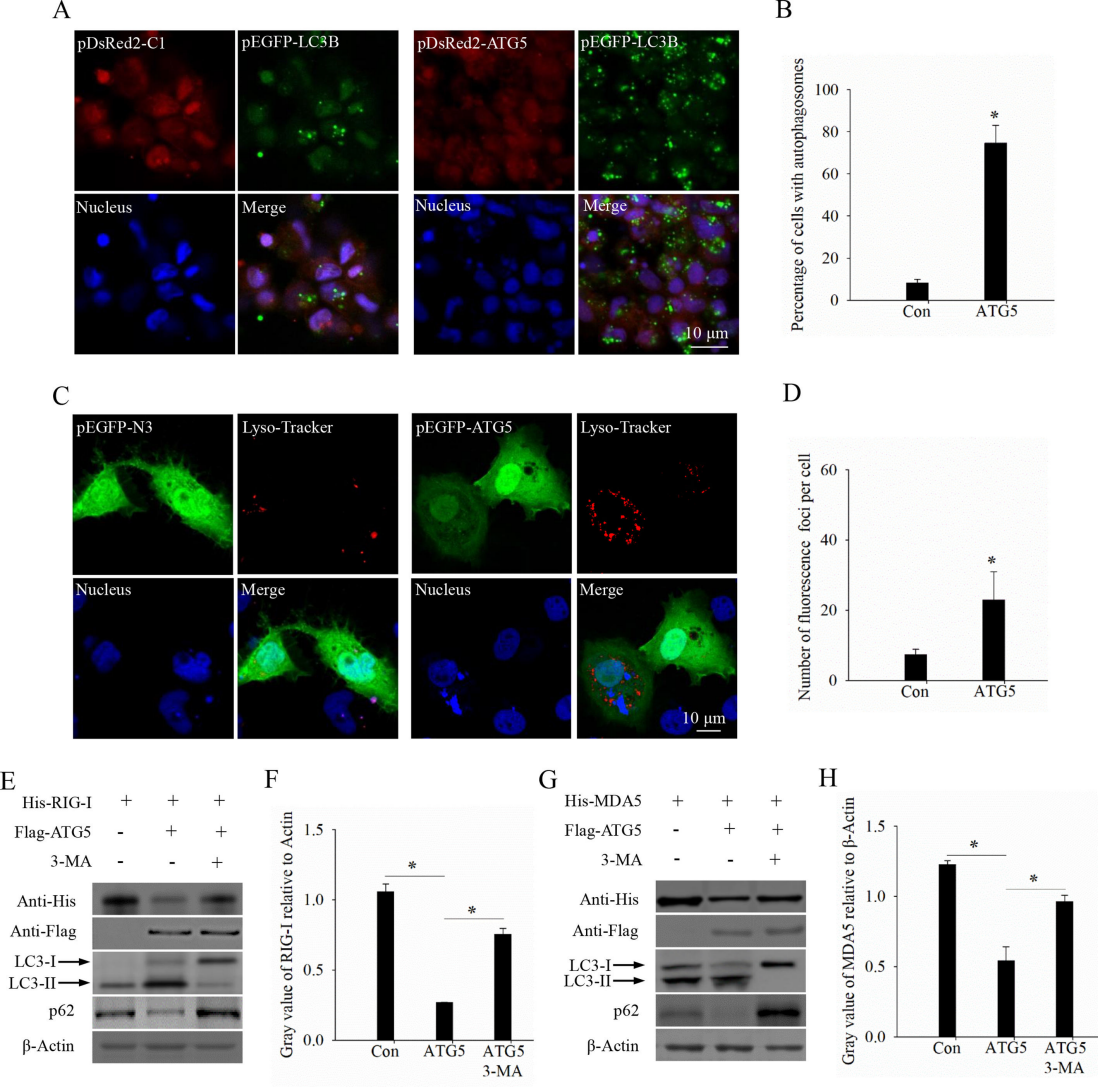
ATG5 promotes autophagy to degrade RIG-I and MDA5. (**A**) Confocal microscopy analysis of the LC3-II-labeled autophagosomes in ATG5- or empty vector-transfected cells. Cells were co-transfected with pEGFP-LC3B and pDsRed2-ATG5 or empty vector for 24 h and then harvested for fluorescence observation. Scale bars = 10 µm. (**B**) Quantitative analysis of the percentage of cells with GFP-LC3 puncta labeled autophagosomes. (**C**) Confocal microscopy analysis of the lysosome activities in ATG5- or empty vector-transfected cells. Cells were transfected with pEGFP-ATG5 or empty vector and then incubated with LysoTracker to stain the lysosomes. Scale bars = 10 µm. (**D**) Quantitative analysis of the number of fluorescence foci per cell. (E–H) ATG5 promotes the degradation of RIG-I and MDA5 in an autophagy-dependent manner. ATG5 was co-transfected with RIG-I (E and F) or MDA5 (G and H) into cells in the absence or presence of 3-MA and then harvested for western blotting analysis (**E, G**) and gray value calculation (**F, H**).

We also investigated whether autophagy induced by ATG5 is involved in the degradation of RIG-I and MDA5, as ATG5 is one of the core genes regulating the autophagy process in both mammals and fish (24, 25). As predicted, ATG5 overexpression significantly increased the number of GFP-LC3 puncta labeled autophagosomes compared to the control group (Fig. 7A and B). Moreover, lysosomal activity, which is closely related to the autophagy process, was higher in the ATG5 overexpression group (Fig. 7C and D). Next, we examined the effect of autophagy induced by ATG5 on the protein levels of RIG-I and MDA5 during GCRV infection. CIK cells were co-transfected with ATG5 and either RIG-I or MDA5 in the presence or absence of the autophagy inhibitor 3-MA, and then sampled for WB analysis. The results revealed that ATG5 promoted autophagy, as evidenced by an increased level of LC3-II and a decreased level of p62, while also suppressing the protein levels of RIG-I and MDA5 in the absence of 3-MA. However, in the presence of 3-MA, we observed a decreased level of LC3-II and an increased level of p62, suggesting the ATG5-promoted autophagy level was inhibited. Moreover, the degradation of RIG-I and MDA5 mediated by ATG5 was also blocked in the presence of 3-MA (Fig. 7E through H). Taken together, these results suggest that ATG5 promotes autophagy to degrade RIG-I and MDA5.

### ATG5 reduces IRF7 phosphorylation by degradation of RIG-I and MDA5

**Fig 8:**
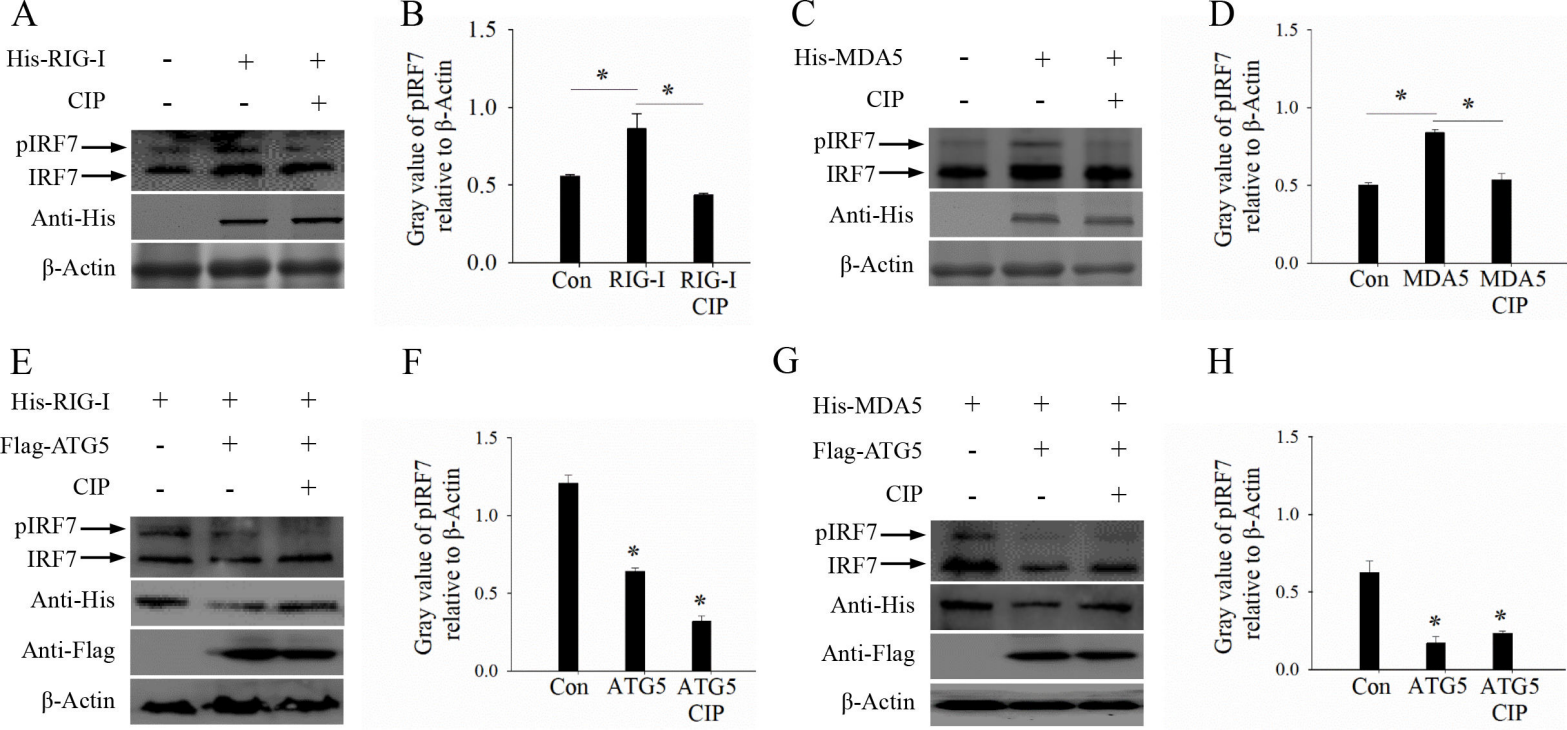
ATG5 reduces IRF7 phosphorylation by degradation of RIG-I and MDA5. (A–D) RIG-I and MDA5 overexpression promoted IRF7 phosphorylation. Cells were transfected with RIG-I (A and B) or MDA5 (C and D) or empty vector and infected with GCRV in the absence or presence of CIP. Then cells were harvested, and the protein expression and phosphorylation of IRF7 were determined by western blotting analysis and (**A, C**) and gray value calculation (**B, D**). (E–H) ATG5 blocked RIG-I and MDA5-mediated IRF7 phosphorylation. Cells were transfected with RIG-I (E and F) or MDA5 (G and H) or empty vector and infected with GCRV in the absence or presence of ATG5 or CIP. Then cells were harvested, and the protein expression and phosphorylation of IRF7 were determined by western blotting analysis and (**E, G**) and gray value calculation (**F, H**).

Phosphorylated IRF7 is a key signal transduction molecule in the RLR signaling pathway, playing a crucial role in activating IFN signaling during GCRV infection (26). In this study, we found GCRV-induced IRF7 phosphorylation was significantly enhanced in the presence of RIG-I and MDA5. In contrast, the RIG-I and MDA5-mediated phosphorylation of IRF7 was blocked by calf intestinal alkaline phosphatase (CIP) (Fig. 8A through D), confirming that RIG-I and MDA5 positively regulate the phosphorylation status of IRF7 during GCRV infection. The above-mentioned results suggest that ATG5 promotes the degradation of RIG-I and MDA5 and reduces the phosphorylation of IRF7. Therefore, we hypothesize that ATG5 decreases IRF7 phosphorylation through the degradation of RIG-I and MDA5. To further investigate this hypothesis, CIK cells were transfected with ATG5 and either RIG-I or MDA5 in the presence or absence of CIP, and then sampled for WB analysis. As anticipated, RIG-I or MDA5-induced IRF7 phosphorylation was inhibited by ATG5 or CIP (Fig. 8E through H). Collectively, these findings strongly suggest that ATG5 promotes the degradation of RIG-I and MDA5, which in turn leads to decreased phosphorylation of IRF7 during GCRV infection.

## DISCUSSION

GCRV-induced hemorrhagic disease poses a significant challenge to the aquaculture industry in China. Previous studies have indicated that GCRV infection rapidly triggers immune responses, including pattern recognition receptors (PRRs), the complement system, IFN signaling pathways, inflammatory pathways, apoptosis, and autophagy (10). Despite these responses, mortality remains high in GCRV-infected fish, particularly in yearling grass carp. This suggests that the acute immune-inflammatory response may be closely associated with GCRV infection outcomes. Cytokine storms, characterized by a sharp increase in IFN and inflammatory factors, are major contributors to fatality in patients with various viral infections, such as severe acute respiratory syndrome, Ebola, and COVID-19 (19, 27, 28). Similarly, 3-year-old grass carp that exhibit resistance to GCRV infection show a reduced immune response upon infection. This supports the notion that investigating the negative regulatory factors of the immune response could identify crucial targets for the prevention and management of viral diseases in fish.

Autophagy is an evolutionarily conserved process that depends on the lysosome pathway to degrade and eliminate useless or harmful substrates, maintaining eukaryotic cell homeostasis (29). It is well-documented that autophagy is tightly associated with immune regulation. A deficiency in autophagy causes enhanced immune responses and worsens the symptoms of various inflammatory conditions (30). In contrast, autophagy inducers can ameliorate symptoms of inflammation-associated tissue damage (31, 32). Our previous study demonstrated that autophagy inhibits GCRV replication and protects *C. idellus* kidney (CIK) cells from excessive inflammatory responses after GCRV infection (13). However, the mechanisms remain unclear.

In this study, we observed that the ATG5 expression level varies between 5-month-old and 3-year-old grass carp, playing a critical role in modulating GCRV infection. In older fish, ATG5 appears to promote autophagy, which helps reduce viral replication. However, in younger fish, the immune response becomes dysregulated, potentially due to lower ATG5 expression and reduced autophagic activity. This could contribute to the increased susceptibility of younger fish to GCRV, as observed in the transcriptomic and protein expression analyses. The hypothesis that viruses might degrade ATG5 or modulate autophagy for their benefit is intriguing. Indeed, several viruses, including hepatitis C virus, rice stripe virus, and foot-and-mouth disease virus, have been shown to degrade autophagy-related proteins to enhance their replication (33–35). It is possible that GCRV, like these viruses, could target ATG5 and inhibit its activity, particularly in younger fish, thereby impairing the autophagic response. This would be consistent with our finding that ATG5 expression is decreased in younger fish, where the immune system is either ineffective or dysregulated, unable to induce an effective autophagic response. While our current data do not directly address whether GCRV specifically degrades ATG5, investigating this possibility would be a valuable direction for future research. Additionally, exploring how age-related changes in autophagic capacity influence viral replication could provide deeper insights into host-virus interaction dynamics across different age groups.

IFNs play a pivotal role in the early stages of viral infection, but excessive IFN production can lead to autoinflammation and autoimmune diseases (36, 37). Therefore, the ability to precisely induce and restrict IFN signaling and immune-inflammatory responses is crucial for improving survival rates and preventing immune-mediated pathology following viral infection (38). Hosts typically employ at least three classical strategies to ensure IFN production and restore homeostasis after viral infection. The first strategy involves inhibitory proteins such as NOD2 and TRIM13, which directly associate with PRR molecules, preventing their activation and thus inhibiting IFN signaling (39, 40). The second strategy involves posttranslational modifications of key molecules, including ubiquitination. For instance, several E3 ubiquitin ligases recognize and bind target proteins, catalyzing the transfer of ubiquitin to a lysine residue, leading to proteasome-dependent degradation of PRR proteins like RING finger protein 122 and U-Box Containing Protein 1 (41, 42). The third strategy involves a lysosome-dependent autophagy pathway. Over recent decades, research has shown that autophagy contributes to the homeostatic regulation of IFN signaling by clearing dysfunctional mitochondria and reactive oxygen species, playing a key role in potentiating RLR signaling (43).

Due to the significant downregulation of the RLR signaling pathway in the ATG5 overexpression group, we investigated the relationship between ATG5 and the RLR signaling pathway. Our results showed that ATG5 not only interacts with RIG-I and MDA5, two key genes in the RLR signaling pathway, but also inhibits their induced immune response. Furthermore, we found that the overexpression of ATG5 led to the degradation of RIG-I and MDA5 in a dose-dependent manner. Given that ubiquitination modification, particularly K48-linked polyubiquitination, is a classical mechanism of protein degradation via the proteasomal pathway (42), we investigated whether ATG5-mediated degradation of RIG-I and MDA5 depends on this pathway. Our results indicate that ATG5 facilitates the K48-linked polyubiquitination of RIG-I and MDA5, leading to their degradation via the lysosomal pathway. However, it remains to be determined whether ATG5 acts independently as an E3 ligase or recruits other E3 ligases to catalyze the K48-linked polyubiquitination of RIG-I and MDA5. Since ATG5 is one of the core genes regulating the autophagy process, we also investigated whether ATG5 induces autophagy to degrade RIG-I and MDA5. As expected, the degradation of RIG-I and MDA5 mediated by ATG5 was blocked in the presence of 3-MA, a classical autophagy inhibitor, suggesting that ATG5 promotes autophagy to degrade RIG-I and MDA5.

In conclusion, we demonstrate that the GCRV infection-induced acute immune-inflammatory response plays a crucial role in determining the infection outcome. ATG5 acts as a negative regulator of this GCRV-triggered immune response. Mechanistically, ATG5 promotes K48-linked polyubiquitination and autophagy to degrade RIG-I and MDA5, resulting in decreased IRF7 phosphorylation and subsequent attenuation of the acute immune-inflammatory response during viral infection. Our findings suggest that targeting ATG5 may be a promising strategy for the prevention and control of GCRV.

## MATERIALS AND METHODS

### Cells and virus

Grass carp kidney (CIK) and grass carp ovary cells were incubated at 28°C and maintained in M199 medium supplemented with 10% fetal bovine serum and 1% Penicillin-Streptomycin in a humidified atmosphere with 5% CO_2_. The study employed two subtypes of GCRV, specifically GCRV-I and GCRV-II, previously isolated and identified by our laboratory (18). GCRV-I was used for virus infection in cultured cells, whereas GCRV-II was used for viral challenge experiments in grass carp.

### Fish and viral challenge experiment

Our previous study showed that grass carp older than 3 years are completely resistant to GCRV infection, while those younger than 1 year remain susceptible (15). Therefore, approximately 300 five-month-old (average weight and length: 8 g and 12 cm) and 300 three-year-old grass carp (average weight and length: 2.5 kg and 50 cm) were used in this study. All fish were obtained from the Guan Qiao Experimental Station, the Institute of Hydrobiology, Chinese Academy of Sciences. Before the experiment, the fish were acclimatized in aerated fresh water at 26–28°C for 1 week before processing. For the viral challenge experiment, the 5-month-old grass carp were intraperitoneally injected with GCRV (GCRV subtype II, 2.97 × 10^3^ RNA copies/µL) at a dose of 20 µL/g of body weight, whereas the 3-year-old grass carp were intraperitoneally injected with concentrated GCRV (GCRV subtype II, 2.97 × 10^4^ RNA copies/µL) at a dose of 2 µL/g of body weight. Before injection (0 days) and at 1–5 days after injection, 15 fish from each group were anesthetized and euthanized with MS-222 (100 mg/L), and the spleen samples were collected for further analysis, including RT-qPCR analysis, histological section preparation, and transcriptome sequencing analysis. The remaining fish were carefully monitored, and daily deaths were recorded. The experiment was concluded when no mortality was recorded for seven consecutive days, and the overall survival rate could be calculated.

### Plasmid, antibodies, and reagents

Plasmids pEGFP-ATG5, pDsRed2-ATG5, pCMV-Flag-ATG5, and pMN155-ATG5 were constructed by inserting the ORF sequence of ATG5 into the pEGFP-N3, pDsRed2-C1, pCMV-Flag, and pMN155 vectors, respectively. pCMV-His-RIG-I and pCMV-His-MDA5 were constructed by cloning the ORF sequences of RIG-I and MDA5 into pCMV-His vector. pMC156-RIG-I and pMC156-MDA5 were constructed by cloning the ORF sequences of RIG-I and MDA5 into pMC156 vector. pGL3-IFN1, pGL3-IRF1, pGL3-IRF3, and pGL3-IRF7 were constructed by inserting the corresponding ORFs into pGL3-basic vector, respectively. pEGFP-LC3B was constructed in our previous study and kept in our lab (13). The pMN155 and pMC156 were gifts from Professor Zongqiang Cui (Wuhan Institute of Virology, Chinese Academy of Sciences). pCMV-HA-Ub and pCMV-HA-Ub-K48O were donations from Professor Wuhan Xiao (Institute of Hydrobiology, Chinese Academy of Sciences).

Rabbit polyclonal antibodies against grass carp ATG5 (anti-ATG5), IRF7 (anti-IRF7), and GCRV VP5 (anti-VP5) were prepared in our laboratory. Briefly, the complete ORF sequences of ATG5, IRF7, and VP5 were amplified and ligated into the pET-28b expression vector. The resulting plasmids were transformed into *E. coli* BL21, and then the bacteria were induced with 1 mM IPTG for 10 h at 20°C to express the fusion protein. The fusion proteins were purified using BeyoGold His-tag Purification Resin (Beyotime, China), mixed with an equal volume of Freund’s adjuvant (Sigma-Aldrich, USA), and thereafter used to immunize the rabbit. The serums were collected after immunizing the rabbit three times. Antibodies against LC3B, p62, and β-Actin were purchased from Abcam (Abcam, UK). Antibodies against HA tag, His tag, and Flag tag were purchased from Sigma (Sigma, USA). MG132, rapamycin, 3-MA, and CIP were purchased from Sigma (Sigma, USA) and used at a final concentration of 20 μM, 100 nM, 5 mM, and 10 U/mL, respectively. Lyso-Tracker Red fluorescent probe (1:20,000) was purchased from Solarbio (Solarbio, China).

### Transcriptome analysis

Transcriptome data of the spleen samples that were collected from 5-month-old and 3-year-old grass carp after GCRV infection, as well as the data of ATG5- or empty vector-transfected CIK cells after GCRV infection, were derived from previous studies performed by our lab and deposited in the Sequence Read Archive at the National Center for Biotechnology Information (accession numbers: PRJNA600033, PRJNA597582, and PRJNA597542) (13, 15). These data were used for GO, KEGG, and GSEA enrichment analyses.

### Dual-luciferase activity assay

CIK cells were seeded in 24-well plates overnight and co-transfected with corresponding plasmids and internal control reporter vector (pRL-TK). At 24 h post-transfection, the cells were infected with GCRV for another 24 h and then harvested. Luciferase activity was measured using a Dual-Luciferase Reporter Assay System (Promega, USA) on a Junior LB9509 luminometer (Berthold Detection Systems, Germany). Relative luciferase activity was normalized to the amount of Renilla luciferase internal control. Data are presented as mean (*n* ≥3) ± standard deviation (SD) of three independent experiments.

### Histopathology and fluorescence microscopy

The GCRV-infected grass carp spleen samples were fixed with 4% paraformaldehyde overnight at 4°C. Following dehydration, the samples were embedded in HistoResin (Leica, Germany). Serial sections of 4 µm thickness were cut using a microtome (Leica, Germany), dried on slides at 42°C overnight, stained with hematoxylin and eosin, and mounted in Permount (Fisher) followed by phase contrast imaging with a 100× oil immersion objective lens. Fluorescence microscopy experiments were performed as described previously (13). Briefly, CIK cells were plated on glass coverslips in six-well plates overnight and transfected with the indicated plasmids for 24 h. Subsequently, the cells were washed, fixed with 4% paraformaldehyde, and stained with Hoechst 33342 (Beyotime, China). Finally, the specimens were observed using a confocal microscope (Leica, Germany).

### RT-qPCR and western blotting analysis

Total RNA was extracted with TRIzol methods and reverse transcribed to cDNA using reverse transcriptase (Vazyme, China). The RT-qPCR was performed using an SYBR Green Realtime PCR Master Mix (Vazyme, China), and β-actin was employed as an internal control gene for cDNA normalization. The qPCR reaction system contained 20 μL SYBR Green Mixture, 10 μL Mix, 1 μL cDNA template, 1 μL each primer, and 7 μL deionized water. The thermal conditions were as follows: 95°C for 5 min, 40 cycles of 95°C for 20 s, 60°C for 20 s, and 72°C for 20 s. The relative expression levels were calculated using the 2^−∆∆Ct^ method (44). Data are presented as mean (*n* ≥3) ± SD of three replicates. All primers for the RT-qPCR assays are listed in Table S1.

For western blotting analysis, the cells were lysed in RIPA buffer on ice for 30 min and protein extracts were separated using 15% SDS-PAGE and transferred onto the PVDF membrane. Then the membranes were blocked with 5% nonfat milk powder at room temperature for 1 h and incubated with primary antibody at 4°C for 12 h. After the final wash, PVDF membranes were incubated with a secondary antibody at room temperature for 2 h. Finally, the immunoblot signals were detected using an HRP-DAB Detection Kit (Tiangen, China). Western blotting results were analyzed using ImageJ software.

### RNAi experiments

To investigate the role of ATG5 during GCRV replication, CIK cells were transfected with siRNAs that targeted ATG5 or negative control by using Lipofectamine 3000 (Thermo Fisher Scientific, USA). After transfection for 24 h, the cells were infected with GCRV and harvested at 24 h post-infection. The harvested cells were used for RT-qPCR and western blotting analysis. The siRNAs used in the study were obtained from GenePharma (GenePharma, China), and the sequences are shown in Table S1.

### Statistics analysis

All experiments were performed at least three times. One-way analysis of variance and unpaired two-tailed Student’s *t*-test were used to analyze statistical significance. Data are represented as mean (*n* ≥ 3) ±SD. Statistical significance is depicted with asterisk (* indicates *P* < 0.05).

## Data Availability

The data generated in the study are available in the published article and its online supplemental material.

## SUPPLEMENTAL MATERIAL

Table S1 jvi.00344-25-s0001.docx Primers used in this study.

ASM does not own the copyrights to Supplemental Material that may be linked to, or accessed through, an article. The authors have granted ASM a non-exclusive, world-wide license to publish the Supplemental Material files. Please contact the corresponding author directly for reuse.
