# Supplementary material for: ATG5 negatively regulates grass carp reovirus-induced immune-inflammatory response by degrading RIG-I and MDA5
Source: J Virol. 2025 Jun 2;99(7):e00344-25. doi: 10.1128/jvi.00344-25 (PMC12282135; doi:10.1128/jvi.00344-25)
Supplement: Table S1 — Primers used in this study. [file jvi.00344-25-s0001.docx]

**Table S1. Primers used in this study.**

| **Primers** | **Sequences (5’—3’)** | **Purpose** |
| --- | --- | --- |
| siATG5-1 | GCCUCUCAAAUGGCACUAUTT | siRNA of ATG5 |
| siATG5-2 | GGUCUGCAGAACGAUAAAUTT |  |
| siATG5-3 | CCGCUAUAUUCCCUUUAGATT |  |
| qATG5-F | CCCTACTATCTGCTCCTCCCACG | qPCR of ATG5 |
| qATG5-R | TTCCCTCATACTCAAACCACATCTCC |  |
| qNS1-F | GTCACTGCCCATTGCTTCGC | qPCR of NS1 |
| qNS1-R | GCGGCACGGGATCTGTTGTA |  |
| qNS2-F | GTCGCCACCTCCACTGGTTT | qPCR of NS2 |
| qNS2-R | ACCAGCGCAAACCATAGCCA |  |
| qVP2-F | AGCCGCACAAATCGCTCGTA | qPCR of VP2 |
| qVP2-R | GGTATGAGGCGCGGAGATGG |  |
| qVP4-F | CTCCTCCTTCACAACCACCAC | qPCR of VP4 |
| qVP4-R | AGCGAGCAAGCTCTTCCAGT |  |
| qIL-1β-F | AAGTTCCCGCTTTGGAGAGTA | qPCR of IL-1β |
| qIL-1β-R | GCCACATACCAGTCGTTCAGT |  |
| qIL-6-F | CAGCAGAATGGGGGAGTTATC | qPCR of IL-6 |
| qIL-6-R | CTCGCAGAGTCTTGACATCCTT |  |
| qIL-8-F | AGGTCTGGGTGTAGATCCACGCTG | qPCR of IL-8 |
| qIL-8-R | TTAGTGTGAAAACTAACATGATCTCT |  |
| qIL-12a-F | CTTTGTCGGGGTCCTAATTATGT | qPCR of IL-12a |
| qIL-12a-R | GTGCTTTTGCTTTGATGATGGA |  |
| qBax-F | CAAGGCTATTTCAACCAAGA | qPCR of Bax |
| qBax-R | CCTGTTCCCTGATCCAGT |  |
| qCaspase8-F | GGTAATCTGGTTGAAATCCGTG | qPCR of Caspase8 |
| qCaspase8-R | CCTTGGCAGGCTTGAATGA |  |
| qCaspase9-F | TAGATGACCAGATGGACGCC | qPCR of Caspase9 |
| qCaspase9-R | AAGGTTGAGTAGGACACCAGGAT |  |
| qBid-F | TCCCTGCTGCTCCTTTCC | qPCR of Bid |
| qBid-R | GTCGGTTTGTAATTCTTCGTCAA |  |
| qTNF-α-F | CGGCATTTACTTCGTCTACAGC | qPCR of TNF-α |
| qTNF-α-R | TAGGAATCGGAAATTCGCATAA |  |
| qIRF1-F | CAGCAGCACCATCACACC | qPCR of IRF1 |
| qIRF1-R | AGAAGAATGAGCGAACGG |  |
| qIRF3-F | TCCAGGCCAAGCATACGAA | qPCR of IRF3 |
| qIRF3-R | CCATTTGCAACAGCCATCAT |  |
| qIRF7-F | CGCCTGTGTTCGTCACTCGT | qPCR of IRF7 |
| qIRF7-R | GGTGGTTGGAAAGCGTATTGG |  |
| qIFNI-F | AAGCAACGAGTCTTTGAGCCT | qPCR of IFN1 |
| qIFNI-R | GCGTCCTGGAAATGACACCT |  |
| qRIG-I-F | ACTACACTGAACACCTGCGGAA | qPCR of RIG-I |
| qRIG-I -R | GCATCTTTAGTGCGGGCG |  |
| qMDA5-F | CAGGAGCGACTCTTGGACTATG | qPCR of MDA5 |
| qMDA5-R | AAAGACGGTTTATTTGAATGGAAG |  |
| qLGP2-F | CGTCTACTCGGTGGTGGCT | qPCR of LGP2 |
| qLGP2-R | AAACTCCCTGGGACTCATACTCT |  |
| qMx2-F | ACATTGACATCGCCACCACT | qPCR of Mx2 |
| qMx2-R | TTCTGACCACCGTCTCCTCC |  |
| qISG15-F | CCCCTTTCCAAGTGTTCGTC | qPCR of ISG15 |
| qISG15-R | ATGGTGCTTCCAGATGTGATGT |  |
| qTBK1-F | GAGACATCAAGCCAGGGAAC | qPCR of TBK1 |
| qTBK1-R | AAAACGTGACTCCGATGCTC |  |
| qSTING-F | CTGCCCCTCAACGCTGTGG | qPCR of STING |
| qSTING-R | CGATTCCGTACCCCTGCTATGT |  |
| qIκBα-F | ATTCACGAGGCCAAAGATGC | qPCR of IκBα |
| qIκBα-R | ACAGCCGGCCTTTAGTAACT |  |
| qNF-κB1-F | CCAGGTGCGGTTTTATGAAGATGA | qPCR of NF-κB1 |
| qNF-κB1-R | ATGGCTTGGGTTCGCTCGTTT |  |
| qβ-actin-F | AGCCATCCTTCTTGGGTATG | qPCR of β-actin |
| qβ-actin-R | GGTGGGGCGATGATCTTGAT |  |
| His-RIG-I -F | TCTCGAGCTCAAGCT TCGAATTCTAT  GGACAATAAAGGGACC | RIG-I Overexpression |
| His-RIG-I -R | CAGTTATCTAGATCCGGTGGATCCTCA  GAGCAGCACAAAGTCTCC |  |
| His-MDA5 -F | GACAAGCTTGCGGCCGCGAATTCAATGG  CAGATGACAAGGATGTGCT | MDA5 Overexpression |
| His-MDA5 -R | GATCCTCTAGAGTCGACTGGTACCTCA  GTCACTGGGCGCCGGGATG |  |
| pMC156-RIG-I-F | ATAAAGCTTGGAGGTGGCGGGAGTGGAGGTGGC  GGGAGTATGTACGAGCTGGAAAAGGAG | RIG-I Overexpression |
| pMC156-RIG-I-R | TATGGTACCGTCTCTCAGCGGCCATGTTTGA |  |
| pMC156-MDA5-F | ATAAAGCTTGGAGGTGGCGGGAGTGGAGGTGGC  GGGAGTATGAGTAGTGATCAGGACGC | MDA5 Overexpression |
| pMC156-MDA5-R | TATGGTACCCTCTGTTCTGTGAAATTCACA |  |
